# The German version of the high-performance work systems questionnaire (HPWS-G) in the context of patient safety: a validation study in a Swiss university hospital

**DOI:** 10.1186/s12913-019-4189-8

**Published:** 2019-06-06

**Authors:** Juliane Mielke, Sabina De Geest, Sonja Beckmann, Lynn Leppla, Xhyljeta Luta, Raphaelle-Ashley Guerbaai, Sabina Hunziker, René Schwendimann

**Affiliations:** 1 Institute of Nursing Science, Department Public Health, University of Basel, Basel, Switzerland; 2 Department of Public Health and Primary Care, Academic Center for Nursing and Midwifery, KU Leuven, Leuven, Belgium; 3 Center of Clinical Nursing Science, University Hospital Zurich, Zurich, Switzerland; 4 Departments of Hematology and Oncology, Freiburg University Medical Center, Freiburg, Germany; 5 Department of Medical Communication/Psychosomatic Medicine, University Hospital of Basel, Basel, Switzerland; 6 Medical Faculty, University of Basel, Basel, Switzerland; 7 Chief Medical Office, Patient Safety Office, University Hospital of Basel, Spitalstrasse 22, 4031 Basel, Switzerland

**Keywords:** Igh-performance work systems (HPWS), Safety climate, Patient safety, Content validity, Psychometrics

## Abstract

**Background:**

High performance work systems (HPWSs) are successful work systems in the context of safety climate and patient safety. The 10-item HPWS questionnaire is a validated instrument developed to assess existing HPWS structures in hospitals. The objectives of this cross-sectional study were to translate the English HPWS questionnaire into German (HPWS-G), to rate its content validity, and to examine its psychometric properties.

**Methods:**

Content validity was examined by a panel of 12 physicians and nurses, and I-CVI and S-CVI calculated. For internal consistency, Cronbach’s α and item-scale correlations were determined. Construct validity was measured via confirmatory factor analysis.

A convenience sample of 782 nurses and physicians in a University hospital setting in Switzerland’s German-speaking region was surveyed. Four inclusion criteria were applied: working in intensive care, emergency department or operating room; having daily patient contact; having worked in the current clinical area for more than three months; and more than 40% employment.

**Results:**

A total of 281 questionnaires were completed (response rate: 35.9%). Overall, the 10-item HPWS-G questionnaire showed good content validity (I-CVI = .83–1; S-CVI = .86) and internal consistency (Cronbach’s α = .853). HPWS-G scores correlated significantly with safety climate (*r*_*s*_ = .657, *p* < .01) and teamwork climate (*r*_*s*_ = .615, *p* < .01). The proposed 1-factor model was accepted considering results of applied minimum rank factor analysis; a confirmatory factor analysis indicated an acceptable to good model fit (GFI = .968; CFI = .902; RMSEA = .043).

**Conclusions:**

The HPWS-G showed good psychometric properties. In clinical practice it can be used to assess HPWS practices and for intra- and inter-hospital benchmarking. Some minor adaptions to the wording could be made as well as reassessing the psychometric properties at other clinical sites.

## Background

High performance work systems (HPWSs) are particularly successful in the context of patient safety [1, 2]. Simply put, HPWSs are bundles of work practices, including information sharing, training, and empowerment, that promote employees’ skills, motivation and participation opportunities and result in improved individual or organisational outcomes such as increased patient satisfaction, efficiency, quality of care and patient safety [3–7]. In hospitals, critical work systems such as those in intensive care units, operating rooms, and emergency departments, which are characterised by specialisation, interdependency and high workflow, are also especially prone to adverse events [8]. Under pressure to find ways to keep patients safe, health care researchers, institutions, and policymakers the world over are focusing on safety culture and teamwork in other sectors; and increasingly, they are recognizing, adapting and implementing HPWSs used in high-risk industries such as aviation and nuclear power [8–11].

### Links between HPWSs, safety culture and patient safety

**Fig. 1:**
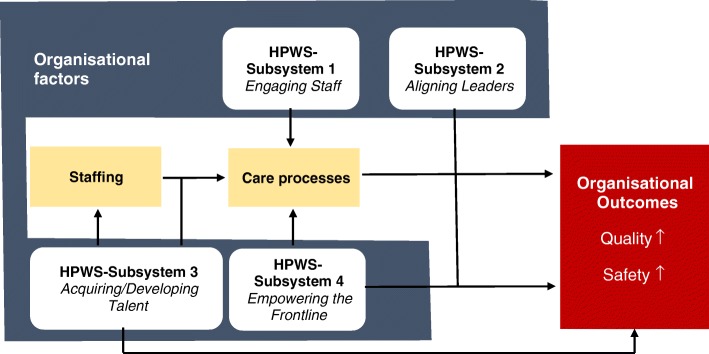
HPWS model in health care; Legend: HPWS model in health care adapted from Garman et al. [3]

Before the current study, one previous conceptual model linked HPWSs to patient safety: Garman et al. (2011) indicated that, as ‘organisational factors’, HPWSs can influence employee and organisation-level outcomes – including patient safety – via multiple pathways [3] (Fig. 1). Two additional critical safety factors affected by HPWS and influencing the same outcomes were staffing and care processes [3, 7]. Chuang et al. (2012) identified three HPWS practices; supervisor support, team-based work practices and flexible work arrangements positively associated with job satisfaction and, when complemented with performance-based incentives, positively associated with frontline health care worker’s perceived quality of care [7]. Other health care related studies revealed that HPWS is positively associated with job satisfaction [12, 13], whereas job satisfaction positively affects perceived quality of care [14, 15]. In systematizing organisational behaviour in terms of three interrelated aspects – culture, structure and processes – Guldenmund’s organisational triangle [16] depicts HPWSs and safety culture as dynamically interrelated. Springing from the ‘underlying values, beliefs and behaviours’ (e.g., at the patient care team and institutional levels), safety culture influences [17, 18] ‘how safety is viewed and treated in an organisation’. And while it is not possible to measure safety culture directly, it can be evaluated in relation to safety climate [9, 10, 17, 19], the surface features of which indicate the characteristics of the underlying safety culture [9].

In contrast to safety culture, safety climate [18] is an aggregation of the ‘shared perceptions of employees about safety relevant aspects’ of their clinical workplaces. By measuring HPWSs, findings supported the association between HPWSs most strongly associated with safety climate [5], e.g. the relationship between HPWSs and higher patient safety scores, lower rates of patient mortality and medication errors [2, 4, 20]. Qualitative study data also indicate that HPWS practices facilitate employees` speak up, an important factor in the context of patient safety culture [21]. Further important factors influencing clinical practice and patient safety include organisational learning and the teamwork climate.

### Measurement of HPWSs

Despite challenges such as construct underrepresentation or construct-irrelevant variance [2], measurement of HPWSs in health care is gaining attention. One promising barometer of HPWS success is Etchegaray et al.’s [5] US-developed and tested 10-item HPWS questionnaire. Based on a literature review and hospital senior executive ratings, this instrument assesses HPWS practice elements such as rewards, employee surveys or job security. The scale showed good psychometric properties (Cronbach’s α = .92); confirmatory factor analysis yielded good construct validity (GFI = .92; CFI = .94*;* RMSEA = .06) [5]. The HPWS questionnaire allows measurements in health care organisations, while developing or selecting HPWS target practices that promote patient safety outcomes via safety culture.

HPWS assessment provides a basis for intra- and inter-hospital benchmarking. Studying their relationships with patient safety outcomes can illuminate factors that potentially provide leverage points for interventions. As each health care context has a unique set of policy and organisational parameters, it is necessary to assess whether observations in the US also hold for other parts of the world. This should be done via tools validated in the target context. As no instrument is yet available to assess and explore HPWS in German speaking health care settings, this study’s two purposes were (1) to translate the original English-language HPWS questionnaire into German (HPWS-G) and rate its content validity; and (2) to examine its psychometric properties, including internal consistency, construct validity and concurrent validity.

## Methods

We used a cross-sectional design, applying a stepwise approach. The first step was to translate the HPWS into German and validate the content of the resulting version (the HPWS-G); the second involved psychometric testing of the HPWS-G. Content validation and psychometric testing adhered to American Educational Research Association (AERA) standards for educational and psychological testing, which include tests of content, internal structure, and relations to other variables [22]. As the completion of the questionnaire was to be anonymous, response processes were not considered. The methods for each objective are separately described below according to the Standards for the Reporting of Diagnostic Accuracy Studies (STARD) statement [23]. To address the study’s overall purpose, i.e., to produce a validated German-language version of the HPWS, we formulated the following objectives (O) and hypotheses (H):

**O1 Translation and content validation of the HPWS-G questionnaire:**

**H1 Evidence based on test content - content validity:**

All items are relevant, appropriate to measure the target HPWS characteristics in the German-speaking Swiss health care setting, and clearly stated.

**O2 Examination of internal consistency, construct and concurrent validity:**

**H2 Reliability - internal consistency:**

The HPWS-G questionnaire demonstrates good internal consistency.

**H3 Evidence based on internal structure - construct validity:**

Empirical Data confirm the proposed 1-factor model of the HPWS-G questionnaire.

**H4 Evidence based on relations to other variables - concurrent validity:**

The HPWS-G questionnaire correlates significantly with the safety climate, teamwork climate, organisational learning, critical incident reporting system (CIRS) practices and patient safety rating subscales.

Using a multistage sampling approach, we first chose a purposive sample from three clinical settings: medical/surgical intensive care units (ICUs), the emergency department (ED), and operating rooms (OR), including surgery and anaesthesiology.

Second, we selected separate samples for objectives 1 and 2 via the inclusion criteria listed below. Both samples included health professionals from a 770-bed University hospital in the German-speaking part of Switzerland.

### Translation and content validity of the HPWS-G questionnaire (O1)

#### Design, setting, sample

The translation process of the original 10-item HPWS questionnaire followed the adapted Brislin translation model [24]. Two Swiss native speakers of German proficient in English translated the questionnaire from English to German, adapted the wording to the Swiss setting. To check their accuracy, a native speaker of English proficient in German translated it back to English. The translation and the original items were compared and checked, leading to a slight revision of wording to ensure comprehensibility. Inconsistencies were discussed in the research team to reach consensus. To examine the HPWS-G’s content validity, a purposive sample of 12 expert health care professionals [25] - six registered nurses (RN) and six physicians - were chosen. Two main inclusion criteria were applied: working in direct patient care and being an experienced clinician (with or without a leadership position) (e.g., clinical nurse specialist, head nurse or senior physician).

### Variables and measurement

Along with 10 HPWS-G questionnaire items (Table 1), the questionnaire included demographic variables such as profession, clinical area, work experience, gender, and age. The experts rated each item’s validity on a 4-point Likert scale (‘not relevant’, ‘somewhat relevant’, ‘quite relevant’, to ‘highly relevant’). Comprehensibility was rated as a ‘yes’/‘no’ response. For both validity and comprehensibility, space was given for additional comments.

**Table 1:** Ten items of the HPWS questionnaire [5]

Item No.	Lable	Employees in my hospital area
1	Skills	… are provided opportunities to learn new skills.
2	Rewards	… are given rewards for doing a good job.
3	Information	… receive necessary information to do a good job.
4	Teamwork	Teamwork is important for providing quality service to patients.
5	Workplace	… are asked how workplace processes can be improved.
6	Appraisal	… receive performance appraisals that help them to improve their performance.
7	Quality	… receive training on quality improvement methods.
8	Job security	… have job security.
9	Survey	… see improvements in this hospital area based on results of employee surveys.
10	Candidate	The best candidate for the job is hired in this hospital area.

### Data collection

After being personally invited to participate, each eligible expert was provided written information about the study’s purpose and a paper copy of the 10-item HPWS-G questionnaire via internal post.

### Data analysis

All 10 item responses were dichotomised as ‘relevant’ (quite or highly relevant) or ‘not relevant’ (somewhat or not relevant) [25]. The item-level content validity index (I-CVI) was calculated by dividing the number of ‘relevant’ ratings for each item by the total number of surveyed experts. To determine the content validity of the overall scale (S-CVI/Ave), all I-CVIs were summed and divided by the number of items [25]. An I-CVI of .78 and S-CVI/Ave values greater than .90 indicated good content validity [26].

### Examination of internal consistency, construct and concurrent validity (O2)

#### Design, setting, sample

A survey was conducted using a sample of virtually all eligible physicians and RNs, working in the study hospital’s medical and surgical ICUs, ED, or OR. Inclusion criteria were daily patient contact, employment in the clinical area for more than three months, more than 40% of full time employment, and having sufficient German language skills to answer the questionnaire.

Ethical approval was obtained from the regional Ethics Committee in July 2017. All participants were informed that the participation was voluntary and fully confidential. Consent was given by answering and returning the questionnaire.

#### Variables and measurement

In addition to the 10 item HPWS-G questionnaire, we added self-developed items assessing the study hospital’s critical incident reporting system practices and overall patient safety. To test validity, we also included subscales from the Safety Attitudes Questionnaire (SAQ) [27] and the Hospital Survey on Patient Safety Culture (HSOPSC) [18] (Table 2). Space was provided for additional comments. Both of the validated and widely used SAQ scales we added assess the safety climate from the health care worker’s perspective. Whereas the SAQ focuses on health care worker perceptions and attitudes regarding patient safety (6 subscales), such as teamwork and safety climate [27, 28], the HSOPSC covers seven unit-level dimensions (e.g., organisational learning), three hospital-level dimensions (e.g., teamwork across hospital units) and four outcome variables (e.g., overall perceptions of safety).

**Table 2:** Variables assessed by the 37-item paper-pencil questionnaire

Variable	Description	Example items	Measurement
HPWS	Ten-item HPWS questionnaire [5] assessing HPWS practices within a given unit.	See Table 1	5-point Likert scale: ‘disagree strongly’ - ‘agree strongly’ (with ‘neutral’); Cronbach’s α = .92 [5]
Teamwork climate (TC)	Six-item subscale from the SAQ [27] (original (English) version [28]) assessing the perceived quality of teamwork and collaboration within a given unit [28].	‘The physicians and nurses here work together as a well-coordinated team.’	5-point Likert scale ‘disagree strongly’ - ‘agree strongly’ (with ‘neutral’); Cronbach’s α = .436–.791 [27]
Safety climate (SC)	Seven-item subscale from the SAQ [27] (original (English) version [28]) assessing perception of how strong and proactive a given unit’s organizational commitment to safety is [28].	‘The culture in this clinical area makes it easy to learn from the errors of others.’	See above
Organisational learning (OL)	Three-item subscale from the HSOPSC [18] (original (English) version [29]) assesses whether mistakes have led to positive changes and changes are evaluated for their effectiveness [30].	‘We are actively doing things to improve patient safety.’	5-point Likert scale ‘strongly disagree’ - ‘strongly agree’ (with ‘neither agree or disagree’); one item ranged from ‘never’ to ‘more than once a month’; Cronbach’s α = .61–.88 [18]
Critical incident reporting system (CIRS)	Three self-developed items assessing the use of CIRSs.	‘I use the CIRS reporting system for the analysis of patient-related events/(near-misses) errors.’	5-point Likert scale ‘strongly disagree’ - ‘strongly agree’ (with ‘neither agree or disagree’)
Patient safety grade (PS)	One self-developed item assesses the overall grade of patient safety within a given unit.	‘What is the overall grade of patient safety in your clinical area?’	10-point visual analogue scale ‘very unsafe’ - ‘very safe’
Demographic data	Seven items assessing each participant’s socio-demographic profile	‘Do you have a management function?’	Dichotomous answer format

### Data collection

Survey data collection took place between 04.10. - 17.11.2017. All eligible RNs and physicians received the 37-item paper-pencil questionnaire via internal mail. A reminder was sent to all participants after two and four weeks, as the period of data collection was extended for two weeks.

### Data analysis

Data were analysed using IBM® SPSS® 24.0.0. For the confirmatory factor analysis (CFA), we used IBM® SPSS® AMOS™ 24.0. Descriptive analysis included each item’s frequencies, percentage, mean, median, interquartile range (IQR) and standard deviation (SD). Data were screened for out-of-range values, homogeneity of variances and univariate outliers by preparing boxplots, histograms and scatterplots [31]. To detect multivariate outliers, Mahalanobis distances were calculated [31]. The level of significance was set at *p* < .05. Before analysis, data were checked for plausibility and comprehensiveness.

#### Internal consistency (reliability) (H2)

First, to determine the degree of inter-item correlation, we generated a correlation matrix including all 10 items. For this to yield a Cronbach’s α of .80 (indicating good internal consistency) an average inter-item correlation of .29 would be necessary [25, 32]. Second, we assessed item-scale correlations, values > .30 correlate very well with the overall scale [32, 33].

#### Construct validity (H3)

To test whether our empirical data confirmed the proposed 1-factor HPWS model, we performed a confirmatory factor analysis. Only non-statistically significant results (*p* < .05) could confirm construct validity [34]. According to Häcker’s [35] recommendations, sample size should be about 200 to 250. Goodness of fit was proved by the goodness of fit index (GFI) (values > .90 indicating good model fit), the root-mean-square residual (RMR), the normed fit index (NFI) (values > .95 indicating reasonable model fit), the comparative fit index (CFI) (values > .95 indicating reasonable model fit), and the root-mean-square error of approximation (RMSEA) (values > .06 indicating reasonable model fit) [31, 35–37]. For potential adjustments of model fit, we finally screened modification indices.

To determine specific indices assessing the dimensionality of the model, we applied the Hull method with minimum rank factor analysis (MRFA) for factor extraction and raw varimax rotating, using FACTOR (Version 10.8.04) [38, 39]. The model was considered unidimensional, if the explained common variance (ECV) index and item explained common variance (I-ECV) ranged between .70 to .85 or above, and mean of item residual absolute loadings (MIREAL) and item residual absolute loadings (I-REAL) were lower than .30 [39].

#### Concurrent validity (H4)

To examine concurrent validity, we needed to compare correlations between ‘HPWS’ and ‘teamwork climate’, ‘safety climate’, ‘organisational learning’, ‘critical incident reporting’, and ‘patient safety grade’. To do so, we generated a correlation matrix, and calculated Spearman’s Rho [34].

## Results

### Translation and content validity of the HPWS-G questionnaire (H1)

The scale-level content validity index (S-CVI) of the 10-item HPWS-G questionnaire was .86, indicating overall good content validity. For the individual items, the content validity (I-CVI) varied between .83 and 1, also indicating good content validity per item. According to the study experts’ ratings and comments, the wordings of five items (‘skills’, ‘information’, ‘teamwork’, ‘appraisal’, and ‘quality’) were slightly revised, or examples added to ensure comprehensibility. After these adaptations, content validity was not re-examined by the experts.

### Examination of internal consistency, construct and concurrent validity (H2–4)

#### Participants

Of 782 questionnaires distributed, 281 (35.9%) were returned. Participants included 165 (58.7%) nurses, 113 (40.2%) physicians, and 3 (1.1%) participants who did not specify their professions. This sample’s socio-demographic data are described in Table 3.

**Table 3:** Socio-demographic characteristics of participants (*N* = 281^a^)

	Total Sample		ICU		ED		OR		Missing	
*Registered Nurses*	*N* = 165		*n* = 58		*n* = 46		*n* = 56		*n* = 5	
Female (%)	70.3		67.2		76.1		71.4			
Age in years, median (IQR)	45.5	(40.0)	43.0	(40.0)	47.0	(36.0)	45.0	(38.0)		
Work experience in years^b^, median (IQR)^c^	17.0	(38.0)	16.5	(38.0)	20.0	(38.0)	17.0	(38.0)		
Work per week (%)										
40–59%	5.5		3.4		13.0		1.8			
60–79%	12.7		15.5		15.2		8.9			
80–100%	80.0		77.6		71.7		89.3			
Leadership function (%)	17.6		19.0		13.0		21.4			
*Physicians*	*N* = 113		*n* = 24		*n* = 20		*n* = 63		*n* = 6	
Female (%)	28.3		33.3		40.0		23.8			
Age in years, median (IQR)	39.0	(35.0)	36.5	(33.0)	34.0	(28.0)	39.0	(34.0)		
Work experience in years^b^, median (IQR)^c^	10	(35.0)	10	(35.0)	7	(29.0)	12	(29.0)		
Work per week (%)										
40–59%	6.2		4.2		15.0		4.8			
60–79%	9.7		16.7		10.0		7.9			
80–100%	83.2		79.2		70.0		87.3			
Leadership function (%)	32.7		20.8		35.0		38.1			

^a^*n* = 3 (1.1) participants could not be assigned to a profession due to missing information; ^b^in this working area (ICU, ED, OR); ^c^IQR = interquartile range

### Response patterns

Conducting a missing values analysis, we found more than 5% missing values in demographic items including age (15.7%) and work experience (5.3%) as well as in the HPWS ‘candidate’ item (6.0%). As Little’s MCAR test yielded a statistically significant result (*p* = .001), we inferred that these data had been missed at random [31]. To conduct a CFA, we replaced missing values with the person-specific mean or, where appropriate, group specific means (items 29^1^ and 30^2^) of the available data.

The Kolomogorov-Smirnov and Shapiro-Wilk tests showed that all items differed significantly from normal (*p* < .001). This was supported by skewness and kurtosis values, as well as Q-Q plots and histograms. Multivariate normality was excluded as the z-value of the Mardia test was 7.383.

Analyses of boxplots, histograms and scatterplots identified two univariate outliers in ‘patient safety grade’. Via the Mahalanobis distance, we detected six (2.5%) multivariate outliers. We calculated regressions for all to highlight what distinguished them from the other cases [31]. In comparison to the main sample, their scores were skewed more extremely, as they often involved the answer option ‘disagree strongly’ or ‘agree strongly’. As we assumed that these cases accurately represented a segment of the population, they were kept in the data set. However, as such cases tend to distort results, robust statistics and methods, such as the median or Bollen-Stine bootstrap, were used [33]. Descriptive analyses of the questionnaire items showed a number of significant response behaviour differences between nurses and physicians, between clinicians with and without leadership functions, and between clinical areas.

### Internal consistency (reliability) (H2)

H2 was supported by a Cronbach’s α of .846. Mean inter-item correlation was .448, and item-scale correlation ranged from 2.77 to 4.62 (Table 4). While ‘teamwork’ and ‘job security’ items showed low discriminating power (.224 and .366), all items were initially retained, as the greatest α increase – by deleting the ‘job security’ item – would be .008. Based on the low factor loading (.053), we decided to delete the ‘teamwork’ item. This produced a corrected Cronbach’s α of .853.

**Table 4:** Internal consistency 10 item HPWS-G questionnaire (reliability); *N* = 281

		Mean	SD	1	2	3	4	5	6	7	8	9	10
*Inter-item correlation*		0.358	0.141										
*Item-scale correlation*				*Inter-item correlation matrix of HPWS-G items*									
1	Skills	4.09	0.761	1.000									
2	Rewards	3.22	0.928	.479	1.000								
3	Information	3.90	0.777	.425	.507	1.000							
4	Teamwork	4.62	0.661	.186	.181	.225	1.000						
5	Workplace	3.21	0.940	.364	.527	.430	.189	1.000					
6	Appraisal	3.24	0.894	.332	.533	.436	.175	.623	1.000				
7	Quality	2.77	1.012	.207	.365	.379	.058	.466	.482	1.000			
8	Job security	3.60	1.164	.111	.235	.268	.169	.225	.254	.305	1.000		
9	Survey	2.92	0.920	.332	.520	.498	.131	.557	.480	.509	.333	1.000	
10	Candidate	3.00	0.986	.434	.481	.499	.208	.424	.462	.365	.305	.429	1.000

### Construct validity (H3)

A CFA based on the remaining nine items initially confirmed the proposed 1-factor model with HPWS as latent factor (*p* (chi^2^) = .215). Fit indices indicated acceptable to good model fit^3^ with chi^2^ = 40.880 (*df* = 27, *p* = .205^4^), GFI = .968, RMR = .025, NFI = .770, CFI = .902, and RMSEA = .043 (90% CI = .008–.068). All factor loadings except for the ‘information’ and ‘job security’ item were significant (*p* < .05) and their size was excellent (> 0.70) [40] (Table 5). However, examination of the modification indices showed significant covariations of certain error variables, suggesting that the model was not unidimensional. To explain these results and improve our factor structure, we therefore applied additional statistics as follows. To examine whether local dependency and unidimensionality were violated, leading to biased parameter estimations, we applied item response theory [41]. After the contribution of the latent trait had been removed, this showed some significant correlations among the items. As the highest correlation between the items ‘information’ and ‘workplace’ was negative (r = −.354, *p* < .000) and other significant correlations were below r < .03, the items’ locally dependency was not clearly confirmed [42]. However, conducted MRFA indicated an ECV index of .888 (95% CI^5^ = .873–.916) and I-ECV values above 0.70, except for the item ‘skills’, suggesting a unidimensional solution. This was supported by a MIREAL of .214 (95% CI^e^ = .178–.251) as well as seven of nine items having I-REAL values lower than 0.30. Furthermore, the generalized H (G-H) indices of both factors, determined for a multidimensional solution, were below .80, with .670 (95% CI = .611–.692) for factor 1 and .764 (95% CI = .730–.778) for factor 2, indicating poorly defined latent variables [39]. Considering these results, for HPWS the one-factor model seems to be most appropriate.

**Table 5:** HPWS-G questionnaire item characteristics (*N* = 281)

		Agree %	Neutral %	Disagree %	Missing %	CFA (1 factor)^a^	
						Loading	*SE* ^b^
1	Skills	80.4	17.4	2.2	–	1.000	–
2	Rewards	38.1	43.1	18.5	0.3	−3.953^‡^	2.031
3	Information	71.9	24.9	3.2	–	1.971^c^	1.072
4	Teamwork	94.3	4.6	1.1	–	–	–
5	Workplace	38.1	41.6	20.3	–	−4.883^‡^	2.438
6	Appraisal	37.0	47.7	15.3	–	−4.139^‡^	2.101
7	Quality	22.4	34.9	41.6	1.1	−5.084	2.574
8	Job security	60.5	22.1	15.6	1.8	−1.221^c^	0.909
9	Survey	23.5	43.4	28.8	4.3	−7.874^‡^	3.825
10	Candidate	27.4	40.9	25.6	6.1	−5.953^‡^	2.935

^a^CFA on 9 items; ^b^standard error; all standardised factor loadings are significant on ^‡^*p* < .05; ^c^not statistically significant

### Concurrent validity (H4)

‘HPWS’ correlated significantly with all five dimensions, i.e., ‘safety climate’ (*r*_*s*_ = .657, *p* < .01), ‘teamwork climate’ (*r*_*s*_ = .615, *p* < .01), ‘organisational learning’ (*r*_*s*_ = .660, *p* < .01), ‘critical incident reporting’ (*r*_*s*_ = .438, *p* < .01), and ‘patient safety grade’ (*r*_*s*_ = .575, *p* < .01). Following Cohen’s [43] directions, we found that the ‘critical incident reporting’ dimension’s effect was medium (*r* > .30); all others’ were large (*r* > .50). These results support our hypothesis. Additional bivariate correlation tests indicated that HPWS was the strongest predictor for safety climate (*r* = .673, *p* < .01), teamwork climate (*r* = .641, *p* < .01), and patient safety grade (*r* = .567, *p* < .01).

## Discussion

This study examined the content validity and psychometric properties of the HPWS-G questionnaire. Content validity of the scale and individual items was confirmed (H1); and the translated questionnaire showed good internal consistency (H2) and concurrent validity (H4). Considering the results of the MRFA, the initially proposed 1-factor model was accepted, indicating acceptable to good model fit (H3). With minor revisions (e.g., wording of items to fit the context), the HPWS-G questionnaire can be used to assess and monitor HPWSs in German speaking hospitals, yielding internationally comparable results.

### Response patterns and demographics

Analysing response patterns revealed differences in response behaviour across professions and clinical areas, as well as across management levels. Regarding safety climate and teamwork climate, such differences have been reported elsewhere [28, 44], highlighting the danger of inferring generalizability across professions or clinical areas.

One of the three items with more than 5% missing values was the HPWS ‘candidate’ item. In that case, we assume that not all respondents could judge whether the best candidate was hired for each job given their position in the team. Considering the above-mentioned inter-group response differences, it might be advisable to use that item only in HPWS questionnaires for respondents with leadership functions. For demographic items, respondents expressed concerns about their anonymity, although we merged the clinical areas, the medical and surgical ICUs, as well as the OR and anaesthesiology.

### Internal consistency (reliability)

The HPWS-G’s Cronbach’s α was lower than that of the original version, but was improved somewhat by deleting the ‘teamwork’ item. Initially, we retained both the ‘teamwork’ and ‘job security’ items despite their low discriminant power, as they play important roles in the HPWS conceptual model [3]. Regarding the ‘teamwork’ item, in combination with its low factor loading in the first factor analysis and the fact that it made a statement^6^ without raising a condition, we decided to exclude it. In fact, the same item had been excluded from the original HPWS questionnaire [5]. The ‘job security’ item also showed low discriminant power – possibly resulting from its strongly right-skewed distribution (which can be improved by reformulating the item [45]); however, its standard deviation was good. Differing national-level employment policies may explain why job security appears less important to the Swiss sample than to those in the US [46].

### Construct validity

The suggested 1-factor HPWS-G model showed an overall acceptable to good fit. The low NFI value might have resulted from underestimation of fit due to the relatively small samples and violations of multivariate normality [47, 48]. Other fit indices such as the CFI overcome these problems [47]. The 1-factor model fits the results of Etchegaray et al. [5], also evidence that the HPWS–G questionnaire distinguishes between safety climate and teamwork climate. Our data did not confirm this, as safety climate and teamwork climate were not included in the CFA. However, HPWS as unidimensional construct is not equivalent to the four HPWS subsystems mentioned in Garman et al.’s [3] model (‘engaging staff’, ‘aligning leaders’, ‘acquiring/developing talent’, ‘empowering the frontline’). One possible reason for this is a lack of a standard HPWS-related terminology, as well as uncertainty about which HPWS patient safety-related practices, and in which combinations, are most promising.

### Concurrent validity

The HPWS-G questionnaire correlates well with safety climate and teamwork climate scales, subscales for patient safety grade, and items on organisational learning and CIRS. In Garman et al.’s [3] conceptual model, HPWS’s influence on patient safety is mediated by staffing and care processes, which could not be shown in our study. Unlike Etchegaray et al.’s findings [5], our analyses indicated that HPWS was a stronger predictor of safety climate than of patient safety grade. Two other studies support this result. First, Guldenmund’s organisational triangle theoretically maintains [16] that safety climate interrelates dynamically with HPWSs and can influence patient safety. Second, Zacharatos et al. [49] showed a mediating effect of safety climate between HPWSs and both safety incidents and personal-safety orientation. Additional qualitative study findings from health care organizations in the U.S. highlight HPWS as crucial element, facilitating speak up, an important factor in the context of safety climate [21].

Transferred to the health care setting, these findings suggest that clinicians should regard the presence of HPWSs as a predictor of safety climate, which in fact impacts outcomes including patient safety (as HPWSs bolster efforts to prevent adverse events [27]). According to the World Health Organization [50], successful adverse event reduction strategies would lead to ‘over 3.2 million fewer days of hospitalisation, 260´000 fewer incidents of permanent disability, and 95´000 fewer deaths per year’ in the European Union alone. Measures reducing adverse events by increasing patient safety and quality focus mainly on the improvement of safety climate [51]. Facilitating development of a robust safety climate – which leads to associated clinical outcomes including, for example, reductions in central line-associated bloodstream infections [52] or patient mortality [20] – is one clear way HPWSs improve patient safety. However, further longitudinal studies will be necessary both to investigate causality between these work systems and safety related outcomes and to support the very encouraging findings reported here and elsewhere.

### Limitations

Considering this study’s known limitations, its results should be interpreted with caution. The convenience sampling procedure and the fairly low response rate may weaken generalizability. However, compared to the validation study of the original English HPWS questionnaire, response rates differ only slightly (35.9% vs. 37.4% [5]). Also, the validation process took place in three high performing clinical areas of the same hospital. Due to language and cultural differences influencing relevance, comprehensibility, and wording, HPWS items may be rated differently by respondents from other units in the same hospital or other hospital settings in Switzerland. As participation was anonymous, evidence of response processes could not be assessed; instead we analysed response patterns. To replace missing values the advantage of chosen procedure (person-specific mean imputation) is that ‘the mean for the distribution as a whole does not change’ [31]. However, variance of the variables could be reduced [31].

## Conclusions

This study provides first evidence supporting the use of a 9-item German-language HPWS practice measurement tool in a Swiss university hospital setting. The German-language HPWS-G questionnaire allows systematic individual-level assessment and monitoring of HPWS practices in clinical settings against the background of patient safety. Our findings allow intra- and inter-hospital benchmarking of HPWSs, encouraging attempts to improve them.

Several minor changes are necessary. Based on psychometric testing results, the wording of the item ‘job security’ needs to be improved. Additionally, as teamwork appears to be important in the context of HPWSs, the deleted ‘teamwork’ item should be reformulated; and the entire 10-item questionnaire cross-validated.

Further research is recommended to test the HPWS-G questionnaire in other clinical units of the study hospital, as well as in other hospital settings. In this way, results assessed via the HPWS-G can be evaluated, informing cut-off values for intra- and inter-hospital benchmarking.

At the interventional level, the HPWS-G can be applied in other settings to assess the relationship between HPWSs and patient safety mediated by safety climate. Finally, we recommend HPWS measurement at the individual (i.e., professional group) or unit level (i.e., clinical area) rather than that of the institution, as tests at these levels best indicate which HPWS practices lead to improved patient safety outcomes in those specific contexts [53].

## Data Availability

The data that support the findings of this study are available from the corresponding author on reasonable request.

## Abbreviations

CFA: Confirmatory factor analysis
CFI: Comparative fit index
ECV: Explained common variance
ED: Emergency department
EFA: Exploratory factor analysis
GFI: Goodness of fit
G-H: Generalized H index
HPWS: High performance work systems
HSOPSC: Hospital Survey on Patient Safety Culture
ICU: Intensive care unit
I-CVI: Item-level content validity index
I-REAL: Item residual absolute loadings
MIREAL: Mean of item residual absolute loadings
MRFA: Minimum rank factor analysis
NFI: Normed fit index
OR: Operating room
RMR: Root-mean-square residual
RMSEA: Root-mean-square error of approximation
RN: Registered nurse
SAQ: Safety Attitudes Questionnaire
S-CVI/Ave: Overall scale-level content validity index
WHO: World Health Organization

## Footnotes

1 How many times in the last six months had CIRS cases been discussed in your clinical area, e.g. in case reviews or team meetings? (‘not at all’ to ‘once a month’).

2 What is the overall grade of patient safety in your clinical area? (Scale from 1 to 10).

3 calculated using Maximum likelihood, *N* = 281.

4 corrected *p*-value performing Bollen-Stine bootstrap.

5 Bootstrap confidence intervals.

6 ‘teamwork is important for providing quality service to patients’.
